# Hypoxia initiates sirtuin1-mediated vascular endothelial growth factor activation in choroidal endothelial cells through hypoxia inducible factor–2α

**Published:** 2012-01-17

**Authors:** Sankarathi Balaiya, Vijay Khetpal, Kakarla V. Chalam

**Affiliations:** University of Florida College of Medicine, Department of Ophthalmology Jacksonville, FL

## Abstract

**Purpose:**

Hypoxia is a critical pathological factor in a variety of retinal diseases, including age-related macular degeneration. It upregulates angiogenic growth factors and promotes neovascularization. Hypoxia changes the cellular redox state and activates class III histone deacetylase sirtuin1 (SIRT1). Activated SIRT1 signals hypoxia inducible factor (HIF)-2α, which transactivates vascular endothelial growth factor (VEGF) and erythropoietin. In this study, we investigated the role of hypoxia induced SIRT1 in choroidal neovascularization in relation to age-related macular degeneration.

**Methods:**

Choroidal endothelial cells (RF/6A) were maintained in a semiconfluent state and hypoxia was induced by exposing the cells to cobalt chloride for 24 h. Induction of hypoxia was confirmed by flow cytometric analysis and the levels of SIRT1 were noted in a hypoxic condition as well in the cells after blocking SIRT1 activity using sirtinol. The role of SIRT1 in the activation of HIF-2α and nuclear factor–κB (RelA/p65) during hypoxia in the presence or absence of SIRT1 was assessed using immunoblot analysis. VEGF levels were quantified using enzyme-linked immunosorbent assay.

**Results:**

Hypoxic induction was confirmed using flow cytometric analysis, which showed cell cycle arrest starting at a 200 µM concentration of cobalt chloride. Hypoxic treatment (200 µM concentration of cobalt chloride) increased SIRT1 levels to 7.8%, which reduced to control level after its activity was inhibited (p<0.05). Activated SIRT1 mediates HIF-2α and nuclear factor-κB (RelA/p65) expression to 4.5 fold and fivefold, respectively, compared to control, and the levels were suppressed following sirtinol treatment (4.1% and 39.3% respectively; p=0.01). Hypoxic treatment increased VEGF levels by 94.9±19.6 pg/ml compared to control levels (25.58±3.58 pg/ml). These levels decreased to 10.29±0.2 pg/ml after blocking SIRT1 activity using sirtinol, compared to control (p<0.01).

**Conclusions:**

Our study results demonstrate that hypoxia mimetic cobalt chloride induces SIRT1 and augments HIF-2α, which activates and releases VEGF.

## Introduction

Ocular neovascularization is associated with a variety of retinal diseases such as age-related macular degeneration (AMD), diabetic retinopathy, and central retinal vein occlusion [1]. Hypoxia and ischemia/inflammation upregulate angiogenic growth factors such as erythropoietin (EPO) and vascular endothelial growth factor (VEGF), and promote neovascularization [2-5]. Regulation of these growth factors is mediated through the activation of transcription factors such as hypoxia inducible factor (HIF) [6].

**Figure 1:**
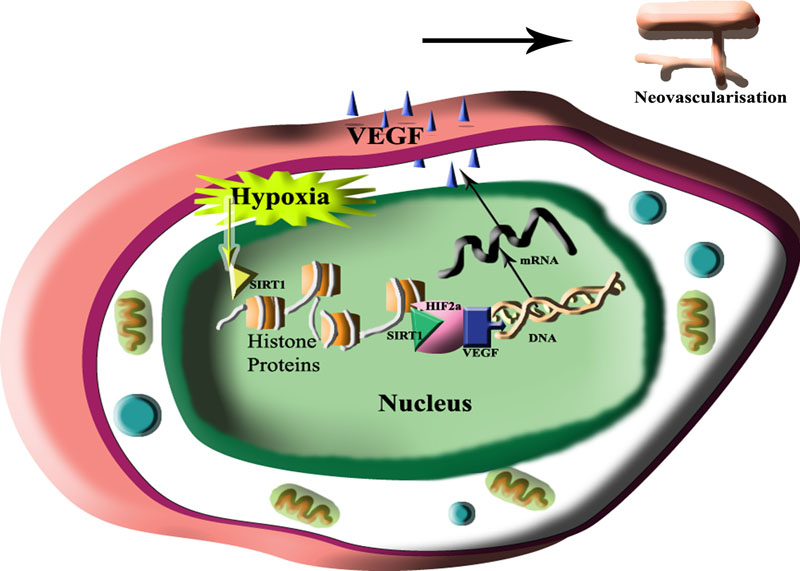
Schematic representation of sirtuin1 role in neovascularisation. During hypoxia sirtuin1 (SIRT1) activates, that binds and transactivate hypoxia inducible factor (HIF)-2α and release vascular endothelial growth factor (VEGF).

Hypoxia alters the cellular redox state, such as in the nicotinamide adenine dinucleotide (NAD^+^/NADH) ratio, and activates stress responsive deacetylase sirtuin1 (SIRT1), a class III histone deacetylase (HDAC) [7-9]. SIRT1 activates HIF-2α and transactivates the downstream genes, including VEGF and EPO, in hypoxic hepatoma and human embryonic kidney cells (Figure 1) [7,10,11].

SIRT1 is expressed in various retinal cells (neural retina, retinal pigment epithelium, and choroid) [9]. However, its role has not been evaluated in hypoxic choroidal endothelial cells (CECs), the precursor of VEGF-mediated choroidal angiogenesis. In this study, we investigated the role of SIRT1 in choroidal angiogenesis in vitro and analyzed the mechanism by which SIRT1 may regulate the initiation of neovascularization.

## Methods

Choroidal endothelial cells (RF/6A; American Type Culture Collection [ATCC], Manassas, VA) were cultured in minimal essential medium (Thermoscientific, Logan, UT) and the media was supplemented with 10% fetal bovine serum (Invitrogen Corp., Carlsbad, CA), 100 U/ml penicillin, and 100 µg/ml of streptomycin (Invitrogen Corp.). Cells were maintained at 37 °C in logarithmic scale in a 75 cm^2^ cell culture flask.

### Induction of hypoxia using cobalt chloride

Hypoxia of CEC was induced by exposing the cells to cobalt chloride (Sigma-Aldrich, St. Louis MO) in serum media, as described previously [12,13], and the induction was confirmed by cytotoxicity and cell cycle analysis. In brief, the dose–response analysis of cobalt chloride was observed at various concentrations (100–700 µM) for 24 h and cytotoxicity was evaluated. Our experiments were performed to evaluate the interaction between HIF-2α and SIRT1; we exposed our cells to cobalt chloride for 24 h.

### Sirtinol

Sirtinol (2-[[(2-Hydroxy-1-naphthalenyl)methylene]amino]-N-(1-phenylethyl)benzamide; C_26_H_22_N_2_O_2_ Tocris Biosciences, Ellisville, MO), a cell permeable inhibitor of SIRT1, was optimized to inhibit sirtuin activity in parallel experiments.

### In vitro cytotoxicity assay

Cell viability was measured by WST-1 (4-[3-(4 iodophenyl)-2-(4-nitrophenyl)-2H-5-tetrazolio]-1.3-benzene disulfonate), a colorimetric assay based on the cleavage of tetrazolium salts to formazan by mitochondrial dehydrogenases in viable cells (Roche, Mannheim, Germany). In complete media, 2x10^3^ cells/well were seeded in 96-well culture plates and maintained to reach 60%–80% confluence (48–72 h) before treatment with cobalt chloride for 24 h. After treatment, cells were incubated with WST-1 solution for 2 h at 37 °C. The plates were read at 440 nm with a reference wavelength at 630 nm using a multidetection microplate reader (BioTek Synergy HT, Winooski, VT). Experiments were triplicated to achieve concordance.

### Cell cycle analysis

Since cytotoxicity of choroidal endothelial cells was noted after 300 µM concentrations, we performed cell cycle analysis to confirm the induction of hypoxia at 100 and 200 µM concentrations of cobalt chloride. This analysis was intended to identify the accumulation of CEC cells in the G2/M phase, giving indirect evidence of hypoxia in CEC.

Cells (10×10^3^) were plated on a six-well plate and maintained to reach 60%–80% confluence. These cells were treated with cobalt chloride at concentrations of 100 and 200 µM for 24 h, collected, and washed in ice-cold PBS before trypsinization, and then diluted to 2.5×10^5^ cells/ml in PBS. After fixation with 70% ethanol for 2 h, cells were digested with DNase-free RNase (100 µg/ml) in PBS. Subsequently, cells were treated with propidium iodide (50 mg/ml in PBS) and incubated for 30 min at room temperature in the dark for DNA staining. Flow cytometry was performed on Cytomics FC 500 (Beckman Coulter, Brea, CA) with CXP V2.2 software. The data were subsequently analyzed with ModFit for cell cycle determination.

Since the induction of stable hypoxia was confirmed at a 200 µM concentration of cobalt chloride, further experiments were performed using this concentration; this is referred to as “hypoxic treatment.”

### Deacetylation activity or evaluation of sirtuin1 activity in cells

To ascertain the role of SIRT1 activity in hypoxic choroidal endothelial cells, intracellular SIRT1 activity was evaluated using an HDAC fluorimetric cellular activity kit (Enzo Life Sciences, Plymouth meeting, PA). This assay is based on cell-permeable Fluor de Lys substrate, which is deacetylated by SIRT1 and yields the deacetylated form of Fluor de Lys substrate, a process dependent on the presence of SIRT1 in the cells. Cells (10×10^3^) were grown on a 96-well microtiter plate and treated with hypoxia (200 µM concentration of hypoxia mimetic cobalt chloride) for 24 h. They were then incubated with 200 µM Fluor de Lys substrate in the presence of 1 µM trichostatin (TSA) at 37 °C, for 4−5 h. The reaction was terminated by adding an ice-cold cell lysis buffer containing Fluor de Lys Developer (Enzo Life Sciences) and 2 mM nicotinamide. Experimental “Time 0” controls were prepared by adding Fluor de Lys substrate with 1 µM TSA and Fluor de Lys Developer (Enzo Life Sciences) with 2 mM nicotinamide immediately to the cell culture plate. Plates were incubated for an additional 15 to 30 min at 37 °C. Fluorescence was determined by reading on a microplate reader (BioTek Synergy HT) with an excitation wavelength of 360 nm and an emission wavelength of 460 nm, and measured as an arbitrary fluorescence unit (AFU). The aforementioned experiment was repeated in presence of sirtinol (200 µM), a competitive inhibitor of SIRT1 activity, to confirm the role of SIRT1 in hypoxia. The levels of SIRT1 were normalized against control and expressed as percentages. Experiments were repeated three times for concordance.

### Preparation of cytosolic and nuclear protein fractions

To determine the SIRT1-mediated expression of HIF-2α and the activation of proinflammatory cytokine nuclear factor (NF)-κB in hypoxic CEC cells, we isolated cytosolic and nuclear protein fractions. Cells (2.5×10^5^) were plated in a 75 cm^2^ culture flask and maintained in an appropriate condition. After hypoxic treatment (200 µM concentration of cobalt chloride), cells were scraped off from the flask and resuspended in 20 ml of Hank’s balanced saline solution and spun at 200× g at 0 °C for 10 min. Cells were washed with Hank’s balanced saline solution followed by spinning at 200× g at 0 °C for 10 min. Cells were suspended in 2 ml of nuclear wash buffer (10 mM HEPES, pH 8.0; 50 mM NaCl, 15% sucrose, 0.1 mM EDTA, 0.5% Triton X-100, 1 mM DTT, 5 mM MgCl_2_, and 1 mM phenylmethylsulfonyl fluoride) and incubated for 10 min at 0 °C. To isolate the cytoplasmic fraction, this cellular suspension was loaded onto 2 ml, 30% sucrose cushion in nuclear wash buffer (without Triton X-100). To isolate the nuclear fraction, following centrifugation at 1,400× g for 30 min (at 0 °C), the pellets were dissolved with a lysis buffer containing 10 mM HEPES, pH 8.0; 600 mM NaCl, 10 mM MgCl_2_, 0.1 mM EDTA, 1 mM DTT, and 5 mM spermidine for 60 min and then centrifuged at 16,000× g for 10 min (at 4 °C). All the fractionation procedures were completed on ice.

### Immunoblot analysis

Protein concentrations of the prepared cytosolic and nuclear extracts were evaluated using Bradford assay solution (Biorad, Hercules, CA). Twenty micrograms of protein samples were run on 8% SDS–PAGE gel and transferred to nitrocellulose membranes, which were blocked in PBS-Tween-20 (PBST) with 5% skimmed milk at 4 °C. The membranes were incubated with primary antibodies for HIF-2α (1:1,000; Abcam, Cambridge, MA) and RelA/p65 of NF-κB (1:2,000; Invitrogen Corporation) in PBS at 4 °C overnight. After washing with PBST, the blots were incubated with horseradish peroxidase-conjugated antirabbit IgG antibodies at a 1:1,000 dilution for 2 h at room temperature. Blots were washed three times in PBST and the proteins were detected with the enhanced chemiluminescence method. Bands were scanned and densitometry was performed to quantify the intensity of signal using ImageJ analysis software. The protein levels were expressed as percentages.

### Vascular endothelial growth factor enzyme-linked immunosorbent assay

We tested whether SIRT1 was involved in induction of angiogenesis through hypoxia by the activation of VEGF. VEGF protein that was released into the conditioned media was measured using enzyme-linked immunosorbent assay (ELISA) after plating 1×10^5^ cells on a six-well culture plate. After treatment, the cell culture media was collected after 24 h of hypoxic treatment at 5% CO_2_ at 37 °C. The concentration of VEGF in the media was measured with an ELISA kit (Invitrogen Corporation) according to the manufacturer’s instructions. The aforementioned experiment was repeated after blocking SIRT1 activity using sirtinol. These VEGF levels were analyzed for correlation to fluorescence levels (AFU) of SIRT1. Experiments were repeated three times for concordance.

### Statistics

Mean and standard deviations (SD) of 3 independent experimental results of each in triplicate were analyzed using GraphPad Instat software (GraphPad Instat3, LaJolla, CA). Means were compared using the two-tailed, unpaired or paired Student *t* test. The correlation between SIRT1 and VEGF values was analyzed using Pearson's correlation coefficient. Statistical significance was accepted for p values of less than 0.05.

## Results

### Induction of hypoxia

**Figure 2:**
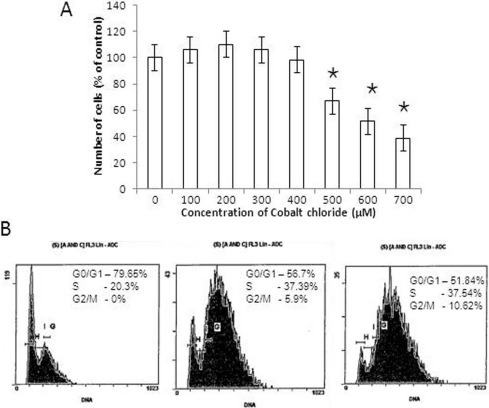
Evaluation of cobalt chloride induced cytotoxicity and the induction of hypoxia. **A**: Hypoxia-induced growth arrest by cobalt chloride at various concentrations (100–700 µM) in choroidal endothelial cells; the concentration of cobalt chloride used for inducing hypoxia is represented on the x-axis and the percentage of viable cells normalized against control on the y-axis. **B**: The effects of hypoxia induced by cobalt chloride on the cell cycle. Choroidal endothelial cells (CECs) were treated with cobalt chloride at various concentrations (0 µM, 100 µM, 200 µM) for 24 h and analyzed for DNA content; representative data are displayed. (Mean+SE; n=3; *p<0.05).

Cobalt chloride treatment did not induce cytotoxicity at doses lower than a concentration of 300 µM, as inferred from WST-1 analysis. At low doses of hypoxia mimetic cobalt chloride (100 and 200 µM), increases in cell viability of 106.0% and 110.0% were noted compared to control cells (p=0.29). At progressively higher doses (300, 400, and 500 µM), cell viability proportionally declined to 102.4%, 98.3%, and 67.0%, respectively, compared to control. We observed 51.7% viability (effective dose [ED]_50_) at a 600 µM concentration of cobalt chloride; this decreased further to 38.6% at a concentration of 700 µM (Figure 2A).

The DNA analysis revealed that 79.65% of control cells were at G_0_/G_1_, 20.35% in the S-phase and none of them at the G_2_/M phase. At a 100 µM concentration of cobalt chloride, the proportion of cells decreased to 56.7% at the G_0_/G_1_ phase, 37.39% at the S-phase, and 5.9% at the G_2_/M phase. At 200 µM, 51.84% cells at the G_0_/G_1_ phase, 37.54% at the S-phase, and 10.62% at the G_2_/M phase was noted (Figure 2B). As the hypoxic challenge mediated through the exposure to cobalt chloride increased, a twofold increase in the proportion of S-phase and G_2_/M phase cells (20.35% to 48%) was noted, which is indirect evidence of the degree of hypoxia (p<0.05).

### Hypoxic treatment induced activation of sirtuin1

**Figure 3:**
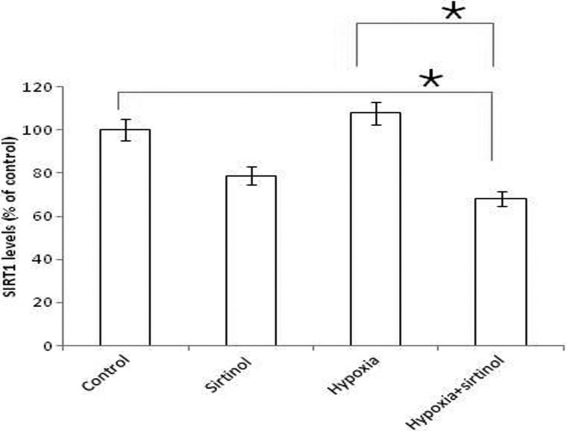
Evaluation of sirtuin1 levels at the cellular level. Choroidal endothelial cells (CECs) were cultured in a 96-well microtiter plate and treated with hypoxia for 24 h. Sirtuin1 (SIRT1) levels were evaluated in hypoxic cells with or without SIRT1 activity being blocked using sirtinol (Mean+SE; n=3;*p<0.05). The x-axis represents the experimental conditions and the y-axis represents the SIRT1 levels in percentage (normalized against control).

In hypoxic cells (200 µM concentration of cobalt chloride), SIRT1 levels increased to 7.8% compared to control. The increase was not statistically significant in comparison to control cells (p=0.4). However, after inhibition with sirtinol, the SIRT1 level decreased 31.6% compared to control. The reduction in SIRT1 levels was statistically significant compared to control cells (p<0.05; Figure 3).

### Increased expression of hypoxia inducible factor–2α and RelA/p65 (nuclear factor–κB) by sirtuin1

**Figure 4:**
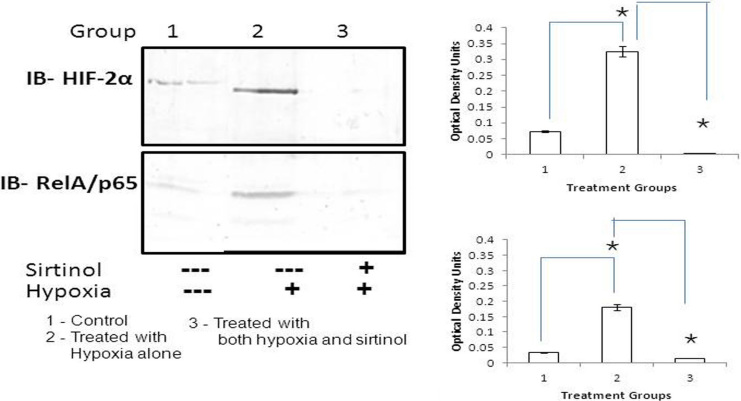
Immunoblot shows hypoxia inducible factor (HIF)-2α and the p65 subunit of nuclear factor κB (NF-κB) protein expression in the nuclear extracts of choroidal endothelial cells during hypoxic condition induced by cobalt chloride. The histogram represents densitometric evaluation of respective protein expression. Choroidal endothelial cells were treated with hypoxia (200 µM concentration of cobalt chloride) in the presence and absence of sirtinol (200 µM). Mean+SE; n=3;*p<0.01.

We evaluated the SIRT1-mediated activation of HIF-2α and proinflammatory cytokine NF-κB in the treated cells. The HIF-2α and NF-κB protein levels were quantified, normalized, and compared against control. In comparison to control cells, we observed a4.5 fold (451%) increase of HIF-2alpha expression in hypoxia-treated cells (200 uM concentration). However, the HIF-2α levels decreased to 4.1% in hypoxia-treated cells after exposed to sirtinol (200 µM). This suppression of HIF-2α expression following the blockage of SIRT1 activity was observed to be statistically significant (p<0.01; Figure 4).

SIRT1 may activate the proinflammatory pathway via the activation of RelA/p65 subunit of NF-κB under hypoxic treatment. In comparison to control cells, we noted a 5.4 fold (542%) increase in the expression of NF-κB (p65) in hypoxic CEC. This expression decreased to 39.3% after blocking SIRT1 activity in hypoxia-treated cells (p<0.01; Figure 4).

### Sirtuin1-mediated activation of vascular endothelial growth factor through hypoxia

**Figure 5:**
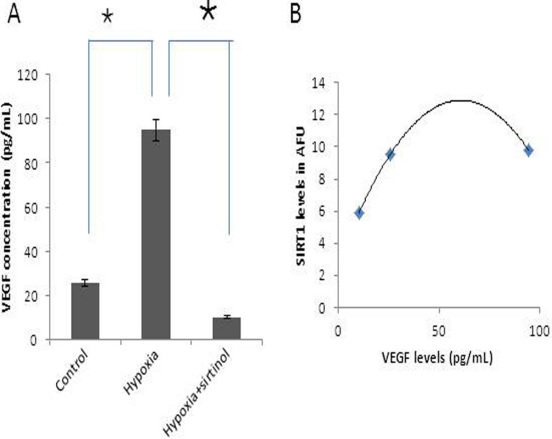
Sirtuin1 (SIRT1) induced vascular endothelial growth factor (VEGF) release. **A**: Evaluation of VEGF levels in the conditioned media after the exposure of choroidal endothelial cells with hypoxia with or without blocking SIRT1 activity (200 uM sirtinol; mean+SE; n=3; *p<0.05) using enzyme-linked immunosorbent assay (ELISA). The x-axis represents the experimental conditions and the y-axis represents the VEGF protein levels in pg/ml. **B**: Correlation of SIRT1 levels measured in the presence of escalating VEGF. The x-axis represents the observed mean VEGF protein levels in pg/mL and y-axis represents their respective SIRT1 levels.

We measured VEGF (121 and 165) in the conditioned media of treated cells that showed 94.9±19.6 pg/mL after the induction of hypoxic treatment (200 µM concentration) compared to a control level of 25.58±3.58 pg/ml. After blocking SIRT1 activity, the VEGF level decreased to 10.29±0.2 pg/ml. This reduction in VEGF level in hypoxia-treated cells after blocking SIRT1 activity was statistically significant compared to control (Figure 5A; p<0.05). In addition, as the SIRT1 increases, VEGF levels linearly correlated in these treatment conditions (Figure 5B; r=0.68).

## Discussion

AMD, a common cause of blindness after the age of 65, is characterized by either the presence of drusen (dry AMD) or VEGF-induced choroidal endothelial cell proliferation with associated leakage (exudative AMD) [14]. In exudative AMD, chronic hypoxia (through HIF-1 and 2) initiates VEGF release and induces choroidal endothelial cell proliferation, an initial step in the formation of choroidal neovascularization. The mechanism of interaction between HIF and the proteins/factors that translate these signals through downstream genes is not well defined. Characterizing these interactions and identifying the role of associated factors are vital for the development of novel therapies that target hypoxia-induced neovascularization.

Histone acetylation and deacetylation play a critical role in cellular events, including gene expression and inflammation [15-17]. Acetylation of conserved lysine residues in the histone tail (by histone acetyl transferases) relaxes the histone-histone and histone-DNA interaction, facilitates transcription factor access to DNA, and enhances gene expression. Deacetylation (by HDACs) suppresses gene expression through the promotion of chromatin condensation [18].

One HDAC member, SIRT1, promotes cell survival by inhibiting stress-induced apoptosis [7,9]. It activates number of proteins like VEGF, EPO, FOXO, p53, and NF-κB [5,15-17]. Silencing SIRT1 activity induces growth arrest and/or apoptosis in human epithelial cancer cells [19], as well as cisplatin-resistant cancer cells [20]. Cambinol, an inhibitor of SIRT1 and SIRT2, promotes apoptosis Burkitt lymphoma xenografts in mice [21]. In Hep3B and HEK293 cells, during hypoxia, SIRT1 deacetylates HIF-2α and increases VEGF expression [5,22]. SIRT1 is expressed in the cornea, ciliary body, lens, retinal pigment epithelium, choroid, and neural retina of mice [9].

In our study, we evaluated the role of SIRT1 in hypoxic choroidal endothelial cells. We induced hypoxia in choroidal endothelial cells with cobalt chloride. Under normoxic conditions, HIF proteins are continuously degraded by binding to the oxygen-dependent degradation domain through ubiquitinylation. Within the oxygen-dependent degradation domain, prolyl hydroxylase enzymes hydroxylate specific proline residues and initiate degradation. Cobalt removes the iron-binding center of the prolyl hydroxylase enzyme, mimics cellular hypoxia, and increases HIF concentration [3,23]. In our study, we observed a hypoxic induction at a 200 µM concentration of cobalt chloride, where flow analysis showed an increase in S and G_2_/M arrest. In transformed cells (mouse embryonic fibroblasts, human gastric carcinoma cells) hypoxia predominantly arrests cells in the S and G_2_/M-phase [23,24].

After induction of hypoxia with cobalt chloride in CEC, we measured SIRT1 levels and compared them to controls. SIRT1 levels were higher in hypoxic cells (1.1 fold) compared to controls (10.25±1.8 AFU; 9.25±1.0 AFU). The role of SIRT1 in hypoxia was confirmed by inhibiting its activity using sirtinol, a chemical inhibitor that selectively inhibits Sir2p transcriptional activity [25]. After treatment with sirtinol, levels of SIRT1 declined to 6.5±1.5 AFU compared to 10.25±1.8 AFU in hypoxia-treated cells (Figure 3).

SIRT1 activates HIF-2α by binding to its C-terminal domain through deacetylation [5]. The activation of HIF-2α was evaluated by assessing HIF-2α protein expression after induction of hypoxia. HIF-2α expression increased to 0.325 optical density units (ODU; 4.5 fold) in hypoxic cells compared to controls (0.072 ODU; p<0.05). After inhibition with sirtinol, HIF-2α expression decreased further (0.003 ODU) and confirmed correlation between SIRT1 and HIF-2α.

SIRT1 regulates the VEGF-A promoter through HIF-2α deacetylation [5]. Based on our results, we hypothesize that elevated SIRT1 levels that result from hypoxic challenge activate HIF-2α and promote VEGF transcription. Increased VEGF levels after hypoxia in the present study and the subsequent decrease after inhibiting SIRT1 activity establish a causal relation between SIRT1 and HIF-2α.

Additionally, in this study, we observed the activation of inflammatory proteins by SIRT1. Activation/deacetylation of the RelA/p65 subunit of NF-κB (at lysine 310) by SIRT1 can transactivate NF-κB-dependent proinflammatory cytokines [26,27]. In our study, expression of RelA/p65 increased to 0.179 ODU (5.4 fold) in hypoxic cells compared to controls (0.033 ODU). After inhibition with sirtinol, RelA/p65 expression was decreased (0.013 ODU); this confirmed the inflammatory role of SIRT1.

In conclusion, our study results demonstrate that hypoxia initiates SIRT1 and augments HIF-2α, which in turn activates and releases VEGF. Our study identifies SIRT1 as an important functional key in determining the cell fate of CECs in hypoxia.
